# How to inherit statistically validated annotation within BAR+ protein clusters

**DOI:** 10.1186/1471-2105-14-S3-S4

**Published:** 2013-02-28

**Authors:** Damiano Piovesan, Pier Luigi Martelli, Piero Fariselli, Giuseppe Profiti, Andrea Zauli, Ivan Rossi, Rita Casadio

**Affiliations:** 1 Bologna Biocomputing Group, University of Bologna, Italy; 2 Department of Biology, University of Bologna, Italy; 3 Department of Computer Science, University of Bologna, Italy; 4 Health Science and Technologies-ICIR, University of Bologna, Italy; 5 BioDec srl, Bologna, Italy

## Abstract

**Background:**

In the genomic era a key issue is protein annotation, namely how to endow protein sequences, upon translation from the corresponding genes, with structural and functional features. Routinely this operation is electronically done by deriving and integrating information from previous knowledge. The reference database for protein sequences is UniProtKB divided into two sections, UniProtKB/TrEMBL which is automatically annotated and not reviewed and UniProtKB/Swiss-Prot which is manually annotated and reviewed. The annotation process is essentially based on sequence similarity search. The question therefore arises as to which extent annotation based on transfer by inheritance is valuable and specifically if it is possible to statistically validate inherited features when little homology exists among the target sequence and its template(s).

**Results:**

In this paper we address the problem of annotating protein sequences in a statistically validated manner considering as a reference annotation resource UniProtKB. The test case is the set of 48,298 proteins recently released by the Critical Assessment of Function Annotations (CAFA) organization. We show that we can transfer after validation, Gene Ontology (GO) terms of the three main categories and Pfam domains to about 68% and 72% of the sequences, respectively. This is possible after alignment of the CAFA sequences towards BAR+, our annotation resource that allows discriminating among statistically validated and not statistically validated annotation. By comparing with a direct UniProtKB annotation, we find that besides validating annotation of some 78% of the CAFA set, we assign new and statistically validated annotation to 14.8% of the sequences and find new structural templates for about 25% of the chains, half of which share less than 30% sequence identity to the corresponding template/s.

**Conclusion:**

Inheritance of annotation by transfer generally requires a careful selection of the identity value among the target and the template in order to transfer structural and/or functional features. Here we prove that even distantly remote homologs can be safely endowed with structural templates and GO and/or Pfam terms provided that annotation is done within clusters collecting cluster-related protein sequences and where a statistical validation of the shared structural and functional features is possible.

## Background

When a new protein sequence becomes available the problem of its annotation poses. Most of our expertise in trying to endow the new sequence with structural and functional features is based on similarity search [1-4]. Methods are mainly based on the knowledge that structure is more conserved than sequence through evolution and that structural alignment is conserved as long as sequence identity (SI) is ≥ 30% over the alignment length. This was observed originally by Chothia and Lesk [5] and once in a while revisited at increasing number of proteins solved with atomic resolution and deposited in the Protein Data Bank (PDB) [6]. The observation is at the basis of one of the most popular method for computing the three dimensional structure of the target on a template, when found, after a sequence similarity search against the PDB [7]. Recently maps of the protein structure space have revealed fundamental relationship between protein structure and function [8]. When a target sequence well aligns with a template of known structure, its functional properties can be derived on the basis of structural conservation. Proteins sharing some 40-60% of sequence identity are likely to share also similar function [9,10].

However a problem is at hand: how to recognize structural and functional templates when sequence identity is below 30%. In this case proteins are categorized to be distantly related to their homologous counterparts, since they may perform the same function, and possibly be endowed with the same structure although sharing very little sequence homology [11,12]. To this purpose methods have developed trying to grasp local sequence conservation by modeling protein conserved structural and functional domains. The most popular is Pfam ([13], http://pfam.sanger.ac.uk). In this case function can be inferred when a protein is significantly retained by a specific Pfam model that is again based on a local sequence-to-profile alignment and its scoring. SUPERFAMILY (http://supfam.cs.bris.ac.uk/SUPERFAMILY), based on hidden Markov models as Pfam, has been recently modified to address specifically the problem of function assignment by including a domain-based Gene Ontology [14].

When function is to be assigned only on the basis of sequence, the problem still remains unsolved, since very little is known on the relationship among sequence similarity and transfer of function [1,9]. Functions can be described with specific terms following the Gene Ontology vocabulary and comprising three main functional branches: Molecular Function (MFO), Biological Process (BPO), and Cellular Component (CCO) [15]. UniProtKB, the largest resource of protein sequences curates automatically annotated protein records ([16], http://www.uniprot.org/help/biocuration). Here annotation integrates previous knowledge on protein structure and function from various sources, when available, again mainly based on sequence similarity search (UniProtKB/TrEMBL). Eventually the records are manually curated (UniProtKB/SwissProt). However out of the over 18 millions sequence entries presently available (Release 2011_12 of 14-Dec-2011), 75% are proteins inferred by homology or predicted whose features in most instances are far from being attributed even with computational methods.

Several methods have been developed to predict protein function from structures and sequences trying to infer features from selected and well annotated sets of proteins by mean of different computational approaches, including machine learning, and generally aiming at integrating different source of information (see for recent reviews [17,18]).

Here we take advantage of the recently released set of proteins selected by CAFA (http://biofunctionprediction.org/) for function prediction in order to discuss how inheritance of annotation can be statistically validated. Validation is indeed an added value to the annotation process, when possible. For this we developed BAR+ [19,20], a non hierarchical clustering annotation procedure that allows different types of annotation by means of a cluster-mediated transfer of annotation. We also show that our method allows a gain of annotation over a direct Pfam prediction and GOA electronic annotation (http://www.ebi.ac.uk/GOA/).

## Databases and methods

### Databases

The test set includes 48,298 sequences made available during the 2011 CAFA experiment (CAFA set, http://biofunctionprediction.org). 41,003 sequences of this set (85% of the CAFA set) could be mapped towards UniProtKB Release 2010_05 (CAFA/UniProtKB set); 96% of the CAFA/UniProtKB set were manually curated (UniProtKB/SwissProt) and 2,047 proteins have also a PDB structure; 13,684 of the set are proteins inferred from homology and predicted. We found that 44,495 sequences of the CAFA set (92% of the CAFA set) could be mapped into BAR+ (CAFA/BAR+ set).

### BAR+

BAR+, the Bologna Annotation Resource, is our annotation system (BAR+ is available at http://bar.biocomp.unibo.it/bar2.0/). BAR+ allows transfer of validated annotation [19,20]. The method relies on the concept that sequences can inherit the same function/s and structure from their counterparts, provided that they fall into a cluster endowed with validated annotations. BAR+ is based on a clustering procedure with the constraint that sequence identity (SI) is ≥ 40% on at least 90% of the pairwise alignment overlapping (Coverage, Cov). Clusters in BAR+, as previously reported [20], allow three main categories of annotation: PDB [with or without SCOP (*)] and GO and/or Pfam; PDB (*) without GO and/or Pfam; GO and/or Pfam without PDB (*) and no annotation. Each category can further comprise clusters where GO and Pfam functional annotations are or are not statistically significant (see below). Depending on the categories of annotation in the cluster and provided that they are statistically validated, all new targets that fall into a cluster can inherit statistically validated annotations by transfer.

For generating BAR+ clusters we analyzed a total of over 13 million protein sequences from 988 genomes and UniProtKB release 2010_05. The BAR+ cluster building pipeline starts with an all-against-all sequence comparison with BLAST in a GRID environment [19]. The alignment results are then regarded as an undirected graph where nodes are proteins and links are allowed only among chains that are 40% identical over at least 90% of the alignment length. All the connected nodes fall within the same cluster; when a cluster incorporates a UniProtKB entry, it inherits its annotations (GO and Pfam terms, PDB structures, SCOP classifications). Within a cluster GO and Pfam terms are statistically validated by means of a procedure that includes P-value evaluation with a Bonferroni correction and estimate of the significance threshold value after a bootstrapping procedure [19]; validated terms are those endowed with P-values< 0.01[19]. Clusters can contain distantly related proteins that therefore can be annotated with high confidence and eventually can also inherit a structural template, if present. In BAR+, when PDB templates are present within a cluster profile HMMs (Hidden Markov Models) are computed on the basis of sequence-to-structure alignment and are cluster associated (Cluster-HMM) [20].

## Results and discussion

### BAR+ contains clusters with statistically validated annotation

70% of the 13,495,736 sequences of BAR+ are collected in 913,762 clusters (the number of sequences in a cluster ranges from 2 to 87,893). Interestingly 87% of the clusters contain sequences whose standard deviation of the protein length is ≤ 5 residues. 1.2% of the clusters, containing 23% of the whole set, contains also PDB structures and is endowed with a cluster specific structural HMM [20]. 30% of the sequences are singletons that eventually can carry along structural and/or functional information.

**Figure 1:**
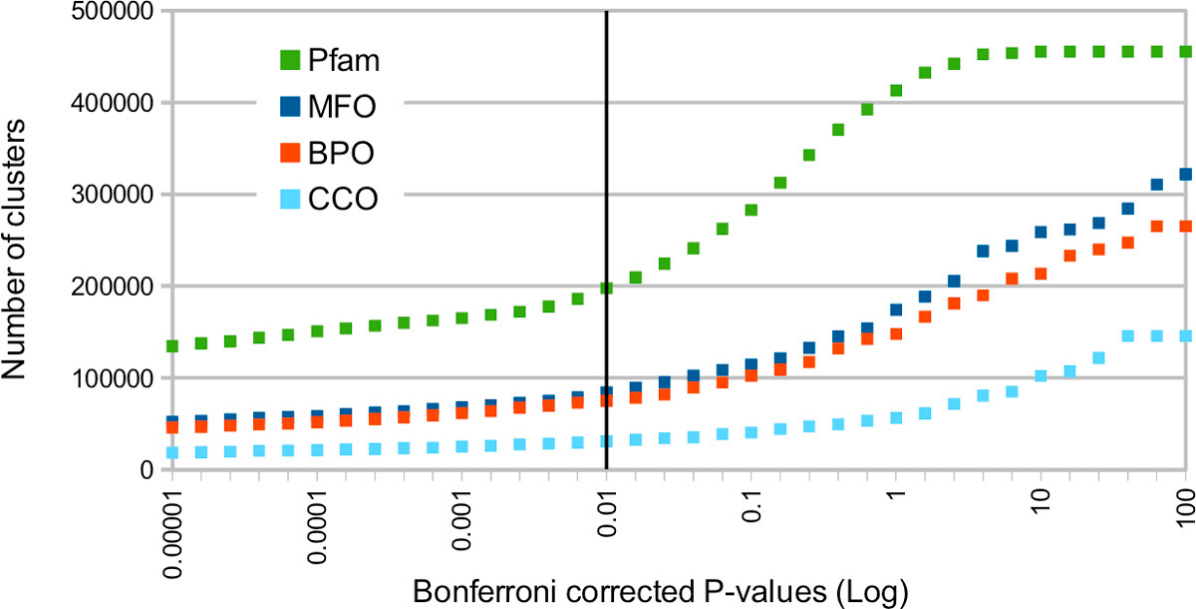
**Discriminating among validated and not validated BAR+ clusters**. The number of clusters containing GO terms of three main roots and Pfam terms is reported as a function of the Bonferroni-corrected P-value. The black vertical line sets the boundary among validated and not validated terms. It can be proven (data not shown) that that a P-value ≤ 0.01 is a discriminative value good enough to discriminate among the real and the random distribution of each type of GO and Pfam terms (for mathematical details see [15]. Green colour: Pfam terms; Blue colour: Molecular Function (MFO); Red colour: Biological Process (BPO); Pale blue: Cellular Component (CCO). For the different curves the number of validated clusters as compared to the total number of BAR+ clusters is: Pfam 197,826/455,309; MFO 84,506/321,748; BPO 75,147/265,164; CCO 31,042/145,677. The total number of cluster with at least a GO validated term is 100,791.

A cluster collects specific Pfam and GO terms directly from the corresponding UniProtKB protein sequence files. Validation of the terms within a cluster is based on a Bonferroni corrected P-value analysis [19]. We performed a statistical evaluation of the P-values by computing the statistical significance of Pfam and GO terms associated to each cluster and by adopting a bootstrapping procedure. By this procedure we determine the threshold at which significance is different from random and we define a P-value equal to 0.01 as the discriminative value for a single term to be validated or not (see also [19]). In Figure 1 the number of clusters is reported as a function of the corresponding Bonferroni corrected P-value for Pfam and the GO terms of the three main roots. The threshold level discriminates among clusters with statistically validated and not validated annotation. 11% of the clusters have one validated GO term allowing in the present version of BAR+ 45% of the total number of sequences (13,495,736) to be included in clusters endowed with validated terms.

Within BAR+, inheritance of validated annotation is possible only when a given sequence after alignment towards BAR+ finds a counterpart whose Sequence Identity (SI) is ≥ 40% over at least 90% of the pairwise alignment overlapping (Coverage, Cov).

### Inheritance of statistically validated annotation

We aligned all the CAFA target sequences against BAR+clusters. More than 92% of the CAFA set was retained by BAR+ (CAFA/BAR+ set), including singletons (stand alone sequences in BAR+). The statistically validated annotations transferred within BAR+ clusters, including Pfam terms and PDB templates (SI≥ 40% and Cov≥ 90%) of the CAFA/BAR+ set are detailed in Table 1. The set of CAFA sequences that received a statistically validated annotation (ALL-O OR Pfam in Table 1) includes 37,516 sequences (77.7% of the CAFA set). The list of predicted proteins is grouped by different target sets including sequences from Eukaryotes, Prokaryotes and "Unknown" organisms. In Table 1 annotations are sorted out by the three different types of GO ontologies and Pfam terms. Values relative to sequences endowed with the union of different ontologies is also shown (MFO OR BPO; ALL-O).

**Table 1:** Annotating the CAFA set with BAR+

	*Cov*	*MFO OR BPO*	*MFO*	*BPO*	*CCO*	*ALL-O*	*Pfam*	*ALL-O OR Pfam*	*PDB°*
*Eukaryotes*	*90%*	20,532	17,389	17,131	16,430	22,733	24,038	26,378	8,054
[32,143]^	*70%*	1,448							

*Prokaryotes*	*90%*	9,660	8,915	8,202	4,723	9,843	10,772	11,088	5,924
[12,295]^	*70%*	224							

*Unknown*	*90%*	36	32	32	10	36	50	50	4
[57]^	*70%*	4							

*Total*		30,228	26,336	25,365	21,163	32,612	34,860	**37,516**	13,982
[44,495]^									2,047*

Cov: Coverage, the ratio of the length of the intersection of the aligned regions on the two sequences and the overall length of the alignment (namely the sum of the lengths of the two sequences minus the intersection length). For both Cov values Sequence Identity (SI) is ≥ 40%. MFO: Molecular Function Ontology; BPO: Biological Process Ontology; CCO: Cellular Component Ontology. ALL-O: number of sequences with predicted MFO *OR *BPO *OR*CCO. Pfam terms. ALL-O OR Pfam: the union of ALL-O and Pfam. °PDB: sequences that inherit a structural template from a cluster HMM within BAR+ [20]. ^ CAFA/BAR+ set sequences from Eukaryotes, Prokaryotes, and Unknown organisms. *Sequences with a corresponding PDB structure.

For sake of exploring the relevance of the alignment length on the annotation system, we decreased the Cov value to ≥ 70%) while keeping SI≥ 40%. In this case the number of annotated CAFA targets increased by only 3% (Table 1), suggesting that the original 90% Cov value together with SI≥ 40% ensures that most of the CAFA set is already retained within validated clusters.

With our method it is also possible to model distantly related targets that fall into a cluster by aligning them to the template/s in the cluster by means of a cluster HMM, as previously described [20]. By this about 25% of the CAFA set inherits also a PDB structural template/s (11,935 sequences, Table 1) and about 50% of these targets share a sequence identity with the template structure of the cluster lower than 30% (12.5% of the CAFA set). Concomitantly the sequence also inherits validated Pfam domains and GO ontologies and this allows a validation of the functional annotation directly on the protein computed structure.

**Figure 2:**
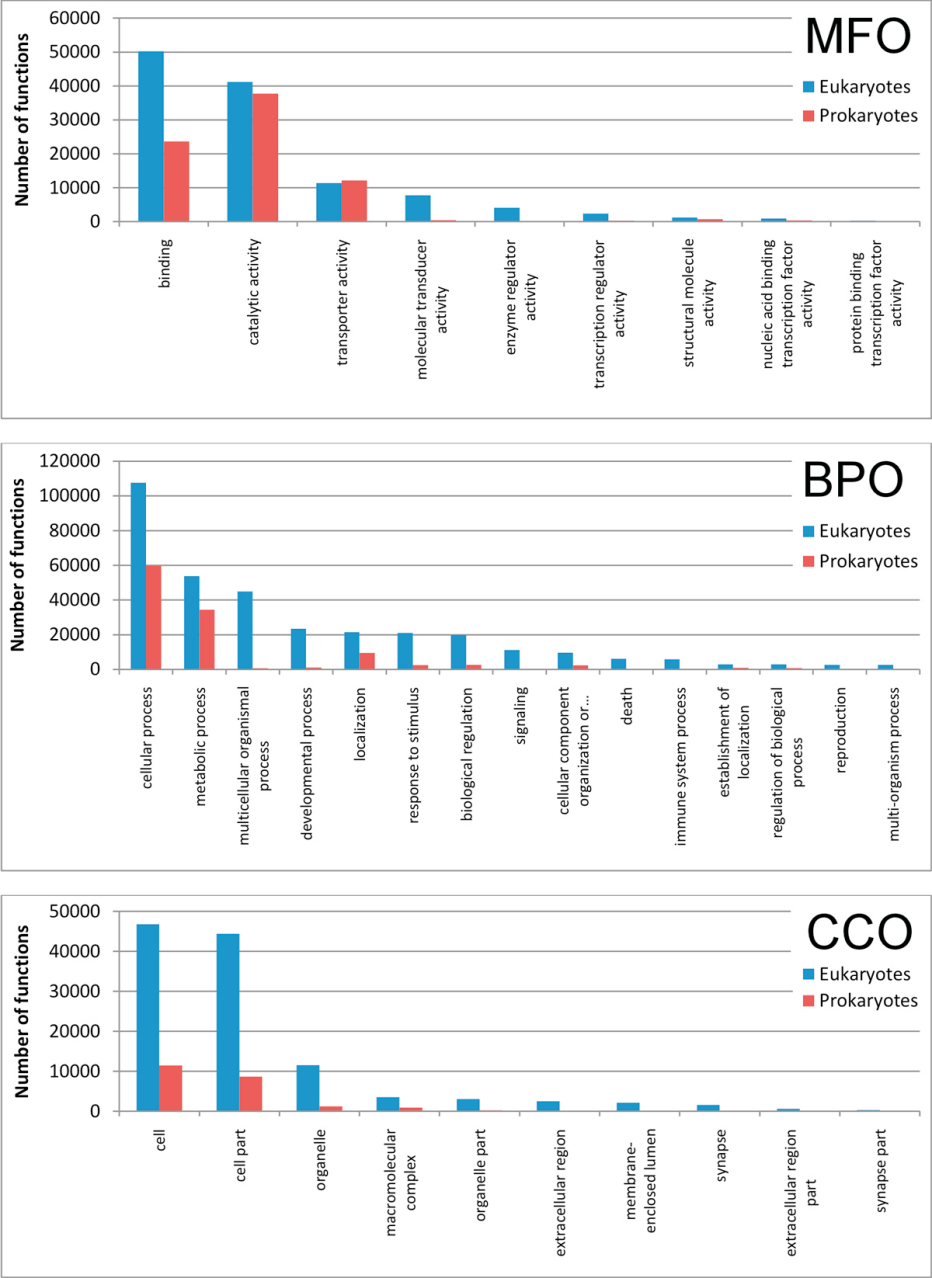
**Statistically validated GO ontologies of the CAFA/BAR+ set**. Histograms of the main statistically validated GO Molecular Functions (MFO), Biological Processes (BPO), Cellular Component (CCO) ontologies are shown after annotation within validated BAR+ clusters. GO terms are included in main categories and listed with respect to Eukaryotes and Prokaryotes.

Statistically validated GO ontologies of the three main roots (MFO, BPO and CCO) are differently distributed among Prokaryotic and Eukaryotic sequences of the CAFA/BAR+ set (Figure 2). Here for sake of simplicity we group all the predicted GO ontologies under the first branches of each principal root. In "Binding" main category "Nucleotide binding" (GO:0000166) and "Protein binding" (GO:0005515) are the most represented in Prokaryotes and Eukaryotes, respectively. In "CatalyticActivity", "Transferase activity" (GO:0016740) and "Hydrolase activity" (GO:0016787) are the most represented in Prokaryotes and Eukaryotes, respectively. The most frequently predicted BPO main category is "Cellular process", with "Cellular biosynthetic process" (GO:0044249) for Prokaryotes and "Cellular macromolecule metabolic process" (GO:0044260) for Eukaryotes. Finally for CCO, the most abundant term both in Prokaryotes and Eukaryotes is "Intracellular" (GO:0005622). The data confirm the variety of statistically validated functional annotations that can be retrieved by adopting BAR+ as an annotation resource and also highlight the main functional features that characterize the proteins of the CAFA set sorted out according to Prokaryotes and Eukaryotes.

**Figure 3:**
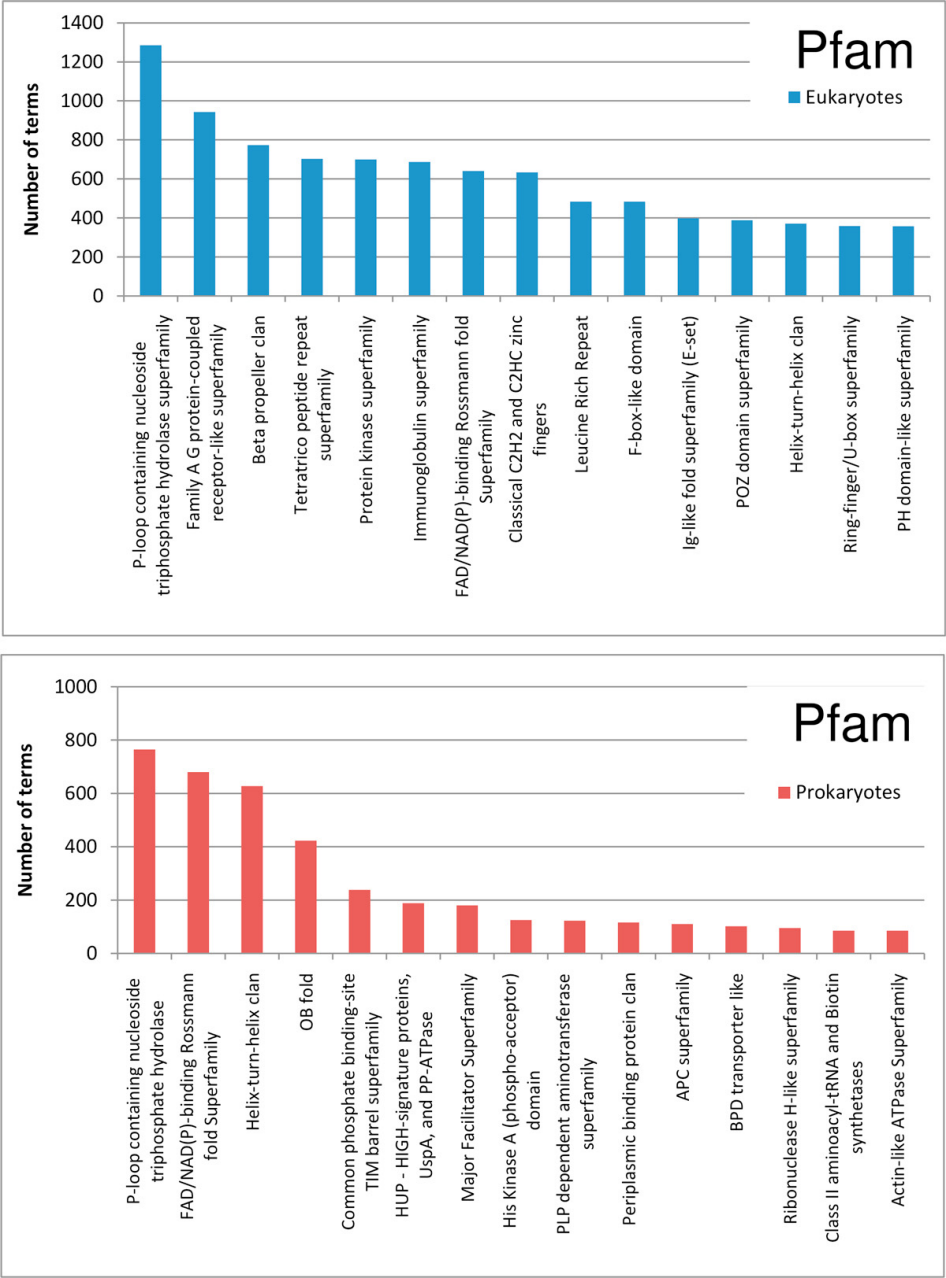
**Statistically validated Pfam terms of the CAFA/BAR+ set**. Histograms of the most populated clans of Pfam terms are shown after annotation within validated BAR+ clusters. A clan is a collection of Pfam-A entries that are judged likely to be homologous [12]. Clans are sorted out discriminating among Prokaryotes (a) and Eukaryotes (b).

In Figure 3 the different validated and inherited Pfam terms are grouped into clans, a collection of Pfam similar entries [12] and shown as a function of the number of sequences from Eukaryotes and Prokaryotes. The most populated clan is "P-loop containing nucleoside triphosphate hydrolase superfamily" (CL0023). Within the clan, the most frequent Pfam domains are Ras family (PF00071) and ABC transporter (PF00005) in Eukaryotes and Prokaryotes, respectively.

### Comparison with direct UniProtKB annotation

34,065 sequences of CAFA/UniProtKB set found a match in 14,747 BAR+ clusters where their annotation is validated (about 71% of the CAFA set) and for 3,659 sequences the number of validated and annotated terms also increases (Table 2: BAR+ validated). The remaining CAFA/UniProtKB sequences (6,938 sequences of which 54% are not annotated) find a counterpart in BAR+ clusters without a statistically validated annotation and are not considered in Table 2. Furthermore, some 15% of the CAFA set (7,295 sequences) does not have a counterpart in UniproKB and they can be aligned towards BAR+ to receive annotation. Out of these, 3,451 sequences receive a statistically validated annotation (Table 2).

**Table 2:** Comparing UniProtKB direct annotation with BAR+ annotation

	CAFA/UniProtKB*		BAR+ Validated°		
	**Sequences**	**Terms**	**Sequences with validated annotation**	**Validated Terms**	**Sequences with new validated annotation**

Total°	34,065	10,628	34,065	13,558	3,659^§^

Pfam^	30,767	5,293	31,190	5,365	423^§^
MFO^	20,790	2,048	21,758	2,698	968^§^
BPO^	19,739	2,719	21,585	4,879	1,846^§^
CCO^	16,503	568	17,589	616	1,086^§^

-	-	-	3,451^#^	5,886^#^	3,451^#^

PDB^+^	2,047^+^	-	13,084^+^	-	11,935^+^

*The CAFA/UniProt KB set (the CAFA sequences that have a UniprotKB file) comprises 41,003 sequences, 3,767 of which do not contain any GO ontology and Pfam terms. °Here the CAFA/UniProtKB subset that can be validated in BAR+ is considered (BAR+validated). The number of sequences and the number of Pfam and GO terms are listed. Sequences that receive new validated terms are also listed according to Pfam, MFO, BPO and CCO. ^# ^Sequences of the CAFA set, out of a total of 7,295 that are not present in UniProtKB and are annotated in BAR+. ^+^Number of sequences that have and also receive in BAR+ a PDB template.

5,215 clusters are also endowed with a cluster HMM, suitable for sequence alignment of the target with the corresponding template/s of 11,935 sequences that by this can inherit also a structure (Table 2). Interestingly 50% of these sequences have a sequence identity to the corresponding template lower than 30%.

### BAR+ web site

For the present analysis, BAR+ was updated by distinguishing two sets of clusters: those that are endowed with a statistically validated annotation (labeled with a yellow star), and those that are not statistically validated. A sequence can inherit annotation from a cluster in a statistically validated manner when upon alignment it falls into a statistically validated cluster; however at the web site for a sequence falling into BAR+ clusters we also provide all the cluster-associated and not validated terms. This is so also when the target aligns towards BAR+ singletons. Each cluster endowed with PDB templates is also endowed with a cluster HMM based alignment that for each sequence falling in the cluster allows building of the corresponding three dimensional protein structure. BAR+ is freely available at http://bar.biocomp.unibo.it/bar2.0/.

## Conclusion

Functional annotation of protein sequences is one of the most important issues in annotation processes. When annotation is done electronically, mainly based on sequence similarity search, a robust validation process can help in the inheritance of Pfam and GO terms by transfer of annotation. Using our cluster-centric BAR+ annotation system and adopting as a test case the recently released CAFA set of sequences, we can annotate 84.9% of the CAFA set, 77.7% of which in a validated manner.

As compared with UniProtKB that annotates with GO and Pfam terms 77.1% of the CAFA set (Table 2), we validate 10,628 terms for 62.9% of the sequences, we increase the annotation for 7.6% of the set with some additional and validated 2,930 terms and annotate without validation the remaining 6.6% of the set.

Considering also that 7.2% of the CAFA set is newly annotated with validation, the gain in annotation within BAR+ is 14.8% with respect to UniProtKB, suggesting again that cluster specificity for a sequence is a necessary filter to inherit functional and structural features from well known proteins.

Furthermore we can endow with structural models some 25% of the whole CAFA set. At least 50% of the proteins that in BAR+ inherit a structural model share a sequence similarity with the template/s less than 30%, indicating that with our procedure also distantly related homologs can be safely annotated.

## Authors' contributions

DP carried out the computational analysis. DP, AZ and IR developed BAR+ under the supervision of PF, PM and RC. GP developed the web site. DP, PF, PM and RC analyzed the data and wrote the manuscript. All the authors have read and approved the final manuscript.

## Competing interests

The authors declare that they have no competing interests.

## Declarations

This article has been published as part of *BMC Bioinformatics *Volume 14 Supplement 3, 2013: Proceedings of Automated Function Prediction SIG 2011 featuring the CAFA Challenge: Critical Assessment of Function Annotations. The full contents of the supplement are available online at URL. http://www.biomedcentral.com/bmcbioinformatics/supplements/14/S3.

## References

[B1] Lesk AM Introduction to Bioinformatics 2008 3 Oxford: Oxford University Press

[B2] Loewenstein Y Raimondo D Redfern OC Watson J Frishman D Linial M Orengo C Thornton J Tramontano A Protein function annotation by homology-based inference Genome Biology 2009 10 207 10.1186/gb-2009-10-2-207 19226439 PMC2688287

[B3] Petryszak R Kretschmann E Wieser D Apweiler R The predictive power of the CluSTr database Bioinformatics 2005 21 3604 3609 10.1093/bioinformatics/bti542 15961444

[B4] Kaplan N Sasson O Inbar U Friedlich M Fromer M Fleischer H Portugaly E Linial N Linial M ProtoNet 4.0: a hierarchical classification of one million protein sequences Nucleic Acids Research 2005 33 D216 D218 15608180 10.1093/nar/gki007 PMC539961

[B5] Chothia C Lesk AM The relation between the divergence of sequence and structure in proteins EMBO J 1986 5 823 826 3709526 10.1002/j.1460-2075.1986.tb04288.x PMC1166865

[B6] Rost B Twilight zone of protein sequence alignments Protein Eng 1999 12 85 94 10.1093/protein/12.2.85 10195279

[B7] Sánchez R Pieper U Melo F Eswar N Martí-Renom MA Madhusudhan MS Mirković N Sali A Protein structure modeling for structural genomics Nat Struct Biol 2000 7 986 990 11104007 10.1038/80776

[B8] Osadchy M Kolodny R Maps of protein structure space reveal a fundamental relationship between protein structure and function Proc Natl Acad Sci USA 2011 108 12301 6 10.1073/pnas.1102727108 21737750 PMC3145735

[B9] Rost B Enzyme function less conserved than anticipated J Mol Biol 2002 318 595 608 10.1016/S0022-2836(02)00016-5 12051862

[B10] Tian W Skolnick J How well is enzyme function conserved as a function of pairwise sequence identity? J Mol Biol 2003 333 863 882 10.1016/j.jmb.2003.08.057 14568541

[B11] Dietmann S Fernandez-Fuentes N Holm L Automated detection of remote homology Curr Opin Struct Biol 2002 12 362 367 10.1016/S0959-440X(02)00332-9 12127456

[B12] Fariselli P Rossi I Capriotti E Casadio R The WWWH of remote homolog detection: the state of the art Brief Bioinform 2007 8 78 87 17003074 10.1093/bib/bbl032

[B13] Finn RD Mistry J Tate J Coggill P Heger A Pollington JE Gavin OL Gunesekaran P Ceric G Forslund K Holm L Sonnhammer EL Eddy SR Bateman A The Pfam protein families database Nucleic Acids Res 2010 38 D211 222 10.1093/nar/gkp985 19920124 PMC2808889

[B14] de Lima Morais DA Fang H Rackham OJ Wilson D Pethica R Chothia C Gough J SUPERFAMILY 1.75 including a domain-centric gene ontology method Nucleic Acids Res 2011 39 D427 34 10.1093/nar/gkq1130 21062816 PMC3013712

[B15] The Gene Ontology Consortium Gene ontology: tool for the unification of biology Nat Genet 2000 25 25 29 10.1038/75556 10802651 PMC3037419

[B16] The UniProt Consortium Ongoing and future developments at the Universal Protein Resource Nucleic Acids Res 2011 39 D214 D219 21051339 10.1093/nar/gkq1020 PMC3013648

[B17] Clark WT Radivojac P Analysis of protein function and its prediction from amino acid sequence Proteins 2011 79 2086 96 10.1002/prot.23029 21671271

[B18] Rentzsch R Orengo CA Protein function prediction--the power of multiplicity Trends Biotechnol 2009 27 210 9 10.1016/j.tibtech.2009.01.002 19251332

[B19] Bartoli L Montanucci L Fronza R Martelli PL Fariselli P Carota L Donvito G Maggi G Casadio R The Bologna Annotation Resource: a non-hierarchical method for the functional and structural annotation of protein sequences relying on a comparative large-scale genome analysis J Proteome Res 2009 8 4362 4371 10.1021/pr900204r 19552451

[B20] Piovesan D Martelli PL Fariselli P Zauli A Rossi I Casadio R BAR-PLUS: the Bologna Annotation Resource Plus for functional and structural annotation of protein sequences Nucleic Acids Res 2011 39 W197 W202 10.1093/nar/gkr292 21622657 PMC3125743

